# The Active Isoforms of MGP Are Expressed in Healthy and Varicose Veins without Calcification

**DOI:** 10.3390/jcm10245896

**Published:** 2021-12-15

**Authors:** Simona R. Gheorghe, Cees Vermeer, Gabriel Olteanu, Ciprian N. Silaghi, Alexandra M. Crăciun

**Affiliations:** 1 Department of Medical Biochemistry, University of Medicine and Pharmacy “Iuliu Hațieganu”, 400349 Cluj-Napoca, Romania; gheorghe.simona@umfcluj.ro (S.R.G.); acraciun@umfcluj.ro (A.M.C.); 2 Cardiovascular Research Institute CARIM, Maastricht University, 6229 ER Maastricht, The Netherlands; cees.vermeer@outlook.com; 3 2nd Surgery Department, University of Medicine and Pharmacy “Iuliu Hațieganu”, 400349 Cluj-Napoca, Romania; golteanu@umfcluj.ro

**Keywords:** varicose veins, matrix Gla protein, calcification, immunohistochemistry

## Abstract

Matrix Gla protein (MGP), a local inhibitor of tissue mineralization, is associated with vascular calcification. Depending on the carboxylation and phosphorylation status, MGP has active conformations, e.g., carboxylated MGP (cMGP) and phosphorylated MGP (pMGP), but also inactive conformations, e.g., uncarboxylated MGP (ucMGP) and dephosphorylated MGP (dpMGP). Our purpose was to assess the presence of all MGP conformations in healthy veins (HV) and varicose veins (VV), concurrently with the analysis of circulating total MGP (tMGP) before and after the surgical stripping of VV. We collected samples from the great saphenous vein, considered as control group, and tissue from VV, designated as VV group. Plasma levels of tMGP were significantly decreased after the surgical removal of the VV (before 59.5 ± 17.2 vs. after 38.1 ± 11.3, *p* < 0.001). By using immunohistochemistry staining, we identified local cMGP and pMGP in the control and VV groups, both without calcification, while ucMGP and dpMGP were absent. cMGP was observed in the nucleus and cytoplasm and pMGP in the nucleus of cells belonging to the tunica media, tunica intima and vasa vasorum. Therefore, the active conformations of MGP (cMGP and pMGP) are prevalent in HV and VV without calcification, affirming their anti-calcifying role in veins.

## 1. Introduction

Throughout the years, researchers have focused on studying the mechanisms and clinical implications of arterial calcification. Although rare [1], venous calcification is also possible and activates a pathway related to osteogenic differentiation of vascular smooth muscle cells (VSMCs) and osteogenic markers, such as osteocalcin and osteopontin, which are expressed in the venous wall of patients with venous reflux [2]. The varicose veins (VV) of the lower limb are superficial, dilated and twisted veins, tributaries to the saphenous or non-saphenous veins [3]. This common condition with a complex and multifactorial etiology, including venous hypertension, extracellular matrix remodeling or venous reflux due to failure of the valves, is sometimes associated with calcification [4]. In VV, matrix Gla protein (MGP), another osteogenic marker, was associated with venous wall calcification [5].

As a paramount local inhibitor of ectopic calcification, MGP has different conformations depending on the post-translational reactions of carboxylation and phosphorylation, namely uncarboxylated MGP (ucMGP), carboxylated MGP (cMGP), dephosphorylated MGP (dpMGP) and phosphorylated MGP (pMGP). Cario-Toumaniantz et al. [5] showed that local ucMGP is abundant in calcified veins and VV, cMGP is prevalent in healthy veins (HV), while increased MGP expression was associated with extracellular matrix (ECM) remodeling and mineralization of VV. The same pattern can be observed in arteries, where local ucMGP is strongly associated with intimal and medial calcification, while cMGP is predominant in healthy arteries [6].

Moreover, in a previous study, we suggested that total MGP (tMGP) synthesized in veins has a contribution to the total pool of circulating MGP [7]. Consequently, to elucidate if MGP has a local contribution as an anti-calcifying mediator at venous level, we decided to evaluate whether the active (cMGP and pMGP) or inactive (ucMGP and dpMGP) conformations are prevalent within the VV and HV and to assess the circulating tMGP levels.

Considering the scarcity of literature related to the prevalence of venous calcification compared to arterial calcification, we hypothesized that mainly the active conformations of MGP could be expressed within the venous walls. The objective of our pilot study was to investigate all MGP conformations in VV and HV as well as to assess their distribution within the venous wall by using immunohistochemistry staining.

## 2. Materials and Methods

### 2.1. Sample Collection and Preparation

The study was conducted on 20 patients diagnosed with VV at the 2nd Surgery Clinic of the Emergency County Clinical Hospital in Cluj-Napoca, admitted for crossectomy, incompetent great saphenous vein stripping and Muller’s phlebectomy. From each patient we collected tissue samples from the great saphenous vein, considered as control group, and from the VV, designated as VV group. Both types of tissue were obtained from the same subjects to eliminate potential influencing factors, such as vitamin K status, smoking status and vitamin K antagonist medication. All tissue samples were fixed in formaldehyde and embedded in paraffin blocks. We excluded patients with vitamin K antagonist treatment. Before enrollment, we obtained written informed consent from each subject, along with their demographics and clinical information. This study was in accordance with the declaration of Helsinki and approved by the Medical Ethics Committee of the University of Medicine and Pharmacy “Iuliu Hațieganu” Cluj-Napoca with the approval code 226/31.05.2018.

### 2.2. Immunohistochemistry Tissue Staining

Consecutive sections of 4 µm thick tissue were cut from the paraffin blocks with the help of a microtome and then mounted on glass slides. After deparaffinization and tissue rehydration, samples were stained for MGP conformations and for the assessment of calcifications by using the von Kossa staining (vK).

For the immunohistochemistry identification of the different MGP conformations, VitaK BV (Maastricht, The Netherlands) provided specific monoclonal antibodies against cMGP (residues 35–54), pMGP (residues 3–15), ucMGP (residues 35–49) and dpMGP (residues 3–15). For antigen retrieval, the rehydrated samples were heated in a 0.2% bath of citric acid for 30 min. We added the primary specific antibodies against cMGP (1.0 µg/mL), pMGP (0.75 µg/mL), ucMGP (0.9 µg/mL) and dpMGP (1.0 µg/mL), diluted in blocking reagent (Roche Diagnostics, Germany). The slides were incubated at 4 °C until the next morning when diluted (1:100) horse radish peroxidase-conjugated rabbit anti-mouse IgG (Dako, Denmark) was applied as the secondary antibody. The antibodies were exposed with NovaRED substrate kit (Vector Laboratories, Burlingame, CA, USA). For the negative control we excluded the primary antibody. Hematoxylin was used for cell nuclei coloration, and samples were preserved with Entallan (Merck, Darmstadt, Germany) mounted coverslips.

The vK protocol was used to identify the presence of tissue calcification. The staining procedure was previously described [8], and all steps were followed accordingly.

A pathology specialist, blinded to the study population, examined the sample slides. Absence of MGP conformation or vK staining was interpreted as negative, and the presence of the two specific stainings in at least one microscopic field was defined as positive. The specific staining is represented by dark red deposits in the analyzed tissue.

### 2.3. Plasma tMGP Assessment

A venous blood sample was collected from each subject in the morning of the surgical procedure and another sample 5 days after the intervention. Both samples were collected in sodium citrate tubes, and the plasma obtained after centrifugation was preserved at −80 °C until analysis.

For the assessment of tMGP plasma levels a sandwich ELISA kit (USCN Life Science Inc., Wuhan, China) was used, and results were read with an Organon Reader 230S (Organon Teknika, Oss, The Netherlands). The detection range for tMGP in plasma was 39–2500 pg/mL. The sensitivity of the assay was 20 ng/L, and our intraday CV was 6.1%.

### 2.4. Statistical Analysis

The population distribution was tested with the Shapiro–Wilk test for normality, and variables were expressed as mean ± standard deviation. The difference between the levels of plasma tMGP before surgery and after surgery was determined with Student’s *t*-test. The difference between the percentage of positive samples for cMGP in media and intima was assessed with the chi-square test. The statistical analysis was performed with the IBM SPSS Statistics 20 software (IBM, Armonk, NY, USA), and statistical significance was reported at *p* < 0.05.

## 3. Results

The anthropometric and clinical characteristics of the subjects are presented in Table 1 as well as the plasma levels of tMGP before and after surgery.

We found that the level of plasma tMGP before surgery is significantly higher than that after surgery (*p* < 0.001).

With respect to local MGP conformations, the immunohistochemical differences between the control group (samples from great saphenous vein) and VV group (samples from VV) are presented in Table 2.

**Figure 1:**
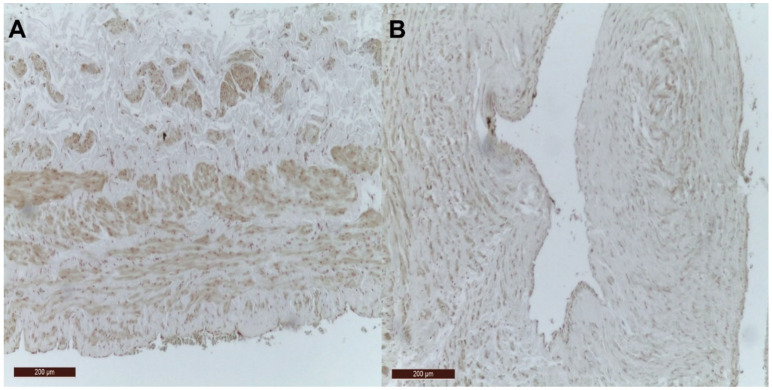
The absence of VK staining in the venous wall. (**A**) VK staining is negative in the wall of the control vein; (**B**) VK staining is negative in the wall of the VV.

Figure 1 illustrates the lack of calcification in control group and VV groups represented by the absence of VK staining.

**Figure 2:**
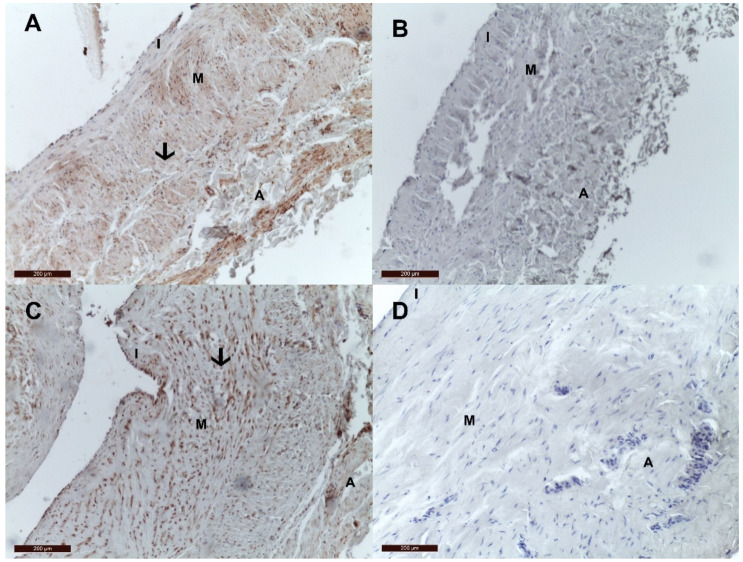
cMGP and ucMGP distribution in control (great saphenous vein) and varicose vein. (**A**) cMGP is present in the wall of the control vein; (**B**) ucMGP is absent in the wall of the control veins; (**C**) cMGP is present in the wall of the VV; (**D**) ucMGP is absent in the wall of the VV. The presence of local cMGP in control and VV is determined by the heterogeneous dark red deposits and highlighted with black arrows. Legend: I, tunica intima; M, tunica media, A, tunica adventitia.

Interestingly, of all MGP conformations, only cMGP and pMGP were present in the venous wall of both control and VV groups, while ucMGP and dpMGP were absent in all samples. Figure 2 depicts the presence of cMGP and absence of ucMGP in the venous wall of a control sample and of a VV sample.

**Figure 3:**
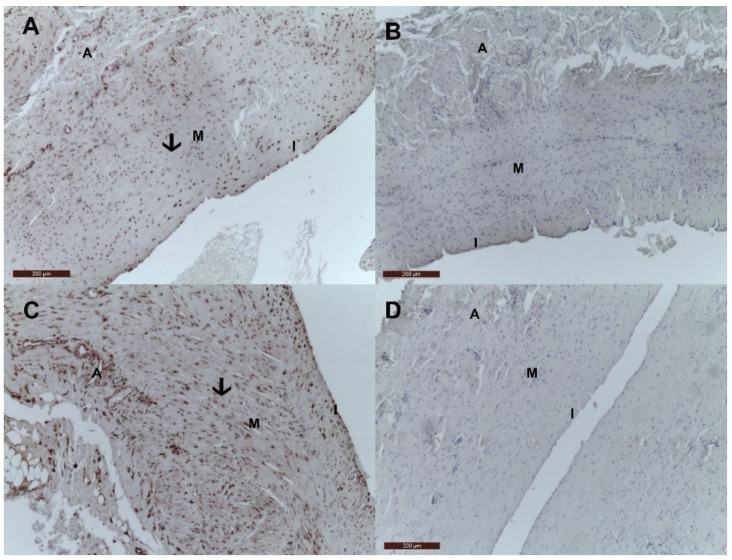
pMGP and dpMGP in control (great saphenous vein) and varicose vein. (**A**) pMGP is present in the wall of the control veins; (**B**) dpMGP is absent in the wall of the control veins; (**C**) pMGP is present in the wall of the VV; (**D**) dpMGP is absent in the wall of the VV. The presence of local pMGP in control and VV is determined by the heterogeneous dark red deposits and highlighted with black arrows. Legend: I, tunica intima; M, tunica media, A, tunica adventitia.

Likewise, Figure 3 illustrates the presence of local pMGP and absence of dpMGP in the wall of a control vein and a VV.

**Figure 4:**
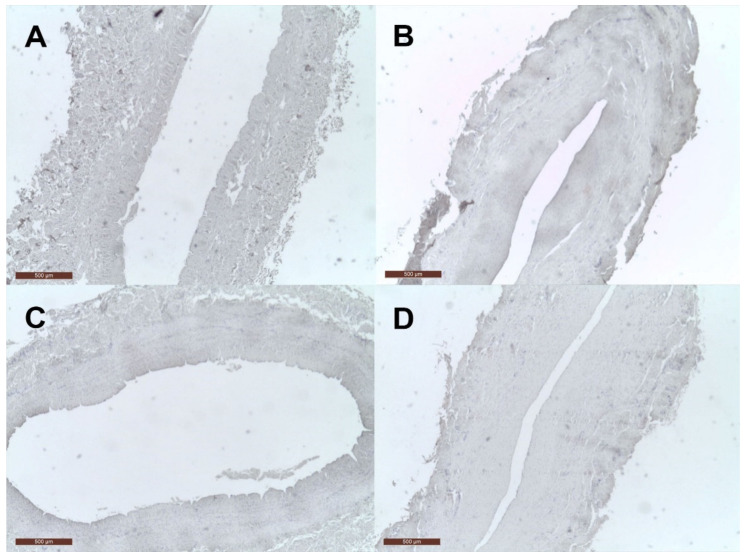
Negative control for cMGP and pMGP in the venous wall. (**A**) negative control for cMGP in the wall of the control vein; (**B**) negative control for cMGP in the wall of the VV; (**C**) negative control for pMGP in the wall of the control vein; (**D**) negative control for cMGP in the wall of the VV.

The negative control samples for cMGP and pMGP in the venous wall are presented in Figure 4.

**Figure 5:**
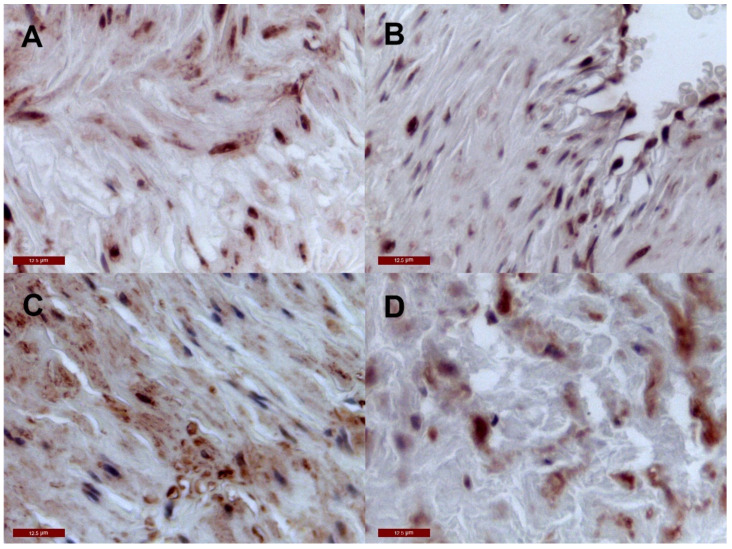
Cellular distribution of pMGP and cMGP. (**A**) pMGP in the nucleus of endothelial and muscle cells of the control veins; (**B**) pMGP in the nucleus of endothelial and muscle cells of the VV; (**C**) cMGP in the nucleus and cytoplasm of endothelial and muscle cells of the control veins; (**D**) cMGP in the nucleus and cytoplasm of endothelial and muscle cells of the VV.

After a comprehensive examination of the tissue samples, we observed that cMGP had a more heterogeneous distribution along the venous wall, being present in the nucleus and the cytoplasm of endothelial and muscle cells, while pMGP was present only in the nucleus, as presented in Figure 5.

**Figure 6:**
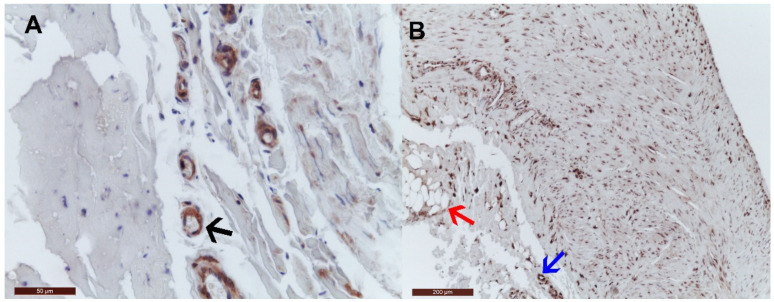
Other distributions of cMGP and pMGP in varicose veins. (**A**) cMGP distribution in the vasa vasorum of the VV (black arrow); (**B**) pMGP distribution in the vasa vasorum (blue arrow) and adipocytes (red arrow) of the VV.

When analyzing the tunica adventitia, we observed that cMGP was positive in the vasa vasorum of the VV, while pMGP was present in the vasa vasorum and the adipocytes. These findings are exemplified in Figure 6.

## 4. Discussion

This is the first study to identify the presence of cMGP and pMGP, as well as the absence of ucMGP and dpMGP, in HV and VV without calcification. Our findings are in contradiction to the previously published study [5] that reported an overexpression of MGP in VV with an abundance of local ucMGP in calcified VV, while cMGP was predominant in HV. They also found that 92% of their VV were calcified, while our samples were negative for calcification.

It is well known that after the vitamin K dependent post-translational carboxylation of glutamate residues and phosphorylation of serine residues, the active conformations of MGP (cMGP and pMGP) inhibit the accumulation of mineral deposits and formation of ectopic calcifications [9]. There are different pathways through which active MGP inhibits vascular calcification [10]. Firstly, being negatively charged, MGP has a high affinity to calcium ions transporting them back to the bone, and secondly, it forms inactive complexes with hydroxyapatite crystals blocking mineral deposit formations [11]. In addition, MGP is known to inhibit the activity of bone morphogenetic protein-2, which has the ability to induce osteoblast differentiation of VSMCs [12]. We believe that in our study the predominance of cMGP and pMGP in veins without calcification confirms the anti-calcification role of the active conformations of MGP. Therefore, we hypothesized that cMGP and pMGP have a protective role against vein calcification, but further studies are warranted to validate this assumption.

A previous study [5] reported the prevalence of cMGP in HV without calcification and in healthy arteries [6], while ucMGP was present in calcified veins and arteries. Vitamin K deficiency impairs the carboxylation of MGP, leading to the accumulation of mineral deposits and the increased production of local ucMGP in tissues [13]. Considering that our research did not identify vein calcification or local ucMGP, we assume that veins are sufficient in vitamin K to activate local MGP, which prevents tissue mineralization.

Cario-Toumaniantz et al. [5] suggested that MGP could be involved in the ECM remodeling of VV through ECM disorganization and VSMCs differentiation and proliferation. Because our research showed that cMGP and pMGP are present in both HV and VV without calcification, it is more likely that MGP is a potent calcification inhibitor in veins.

With respect to its structure, the vein wall is composed of three layers or tunica: intima, media and adventitia. Tunica intima is the innermost area and contains one layer of endothelial cells and connective tissue. Tunica media, the middle layer, consists of VSMCs intermingled with elastic fibers. The outermost layer, the adventitia, provides support and houses the nervous fibers and the vasa vasorum, as well as fibroblasts and VSMC residues. Our study showed that the distribution of cMGP and pMGP in the venous wall was almost identical, both conformations being present in the intima and media layers. The VSMCs from the media secrete MGP, explaining the positive staining in this layer. The presence of MGP in the tunica intima supports the previous finding that MGP is expressed by endothelial cells [14], contributing to vascular stability and integrity.

We found that the distribution of pMGP corresponds to the nucleus, while cMGP was more heterogeneous, being present in the nucleus and cytoplasm. This distribution of cMGP and pMGP indicates that the venous walls may have a contribution to the anti-calcification process in HV and VV.

The active cMGP and pMGP were also present in the adventitial vasa vasorum of the veins. The major role of these small vessels is to provide nourishment to the vascular wall [15]. They are known to contain VSMCs, which are also responsible for MGP synthesis, explaining our findings. Therefore, the additional contribution of the vasa vasorum with the active MGP conformations could contribute to the absence of vein calcification.

Moreover, we identified the presence of pMGP in the perivascular adipocytes. Previous research [16] reported MGP as a novel adipokine, being highly secreted by adipocytes. Adipokines are known to have both anti- and pro-inflammatory roles [17]. With further studies MGP could be considered an integral component of the immune system involved in the local inflammation.

When analyzing circulating MGP, we found significantly lower levels of plasma tMGP after surgery compared to levels before surgery, and likewise for the reference range [18]. It has been demonstrated that MGP is synthesized by VSMCs, which can also be found in the venous wall [5]. The surgical reduction of veins, which simultaneously led to a quantitative decrease in VSMCs, most probably resulted in a significant decrease in circulating tMGP by a third of its initial concentration before surgery. This considerable difference of tMGP levels suggests the contribution of MGP by venous provenance to the global plasma MGP concentration, strengthening our previous findings [7].

The study should be interpreted considering its limitations of a small study population or the lack of information on the status of arterial calcifications. Future studies should focus on comparing circulating and local MGP conformations in HV and VV on a larger cohort.

In conclusion, this is the only study to assess the local presence of all MGP conformations in HV and VV. Moreover, we are the first to report the presence of the active cMGP and pMGP in VV without calcification, confirming their role as a calcification inhibitor not only in arteries, which was demonstrated before, but also in veins.

## Figures and Tables

**Table 1:** Characteristics of study population.

	All (*n* = 20)
Anthropometric and clinical characteristics	
Gender, male/female	7/13
Age, years	46.42 ± 8.59
BMI, kg/m^2^	31 ± 5
T2DM, *n* (%)	2 (10)
RD, *n* (%)	2 (10)
CVD, *n* (%)	4 (20)
HT, *n* (%)	18 (90)
Smokers, *n* (%)	8 (40)
Biochemical analysis	
tMGP, µg/L	
before surgery	59.5 ± 17.2
after surgery	38.1 ± 11.3

Data are presented as mean ± standard deviation or number and percentage n (%), as appropriate. Abbreviations: BMI, body mass index; T2DM, type 2 diabetes mellitus; RD, renal disease; CVD, cardiovascular disease; HT, hypertension; tMGP, total matrix Gla protein.

**Table 2:** Distribution of MGP conformations in the venous wall.

	cMGP	ucMGP	pMGP	dpMGP	vK
**Media**					
All (*n* = 40)	38 (95)	0 (0)	39 (97.5)	0 (0)	0 (0)
Control (*n* = 20)	20 (100)	0 (0)	19 (95)	0 (0)	0 (0)
VV (*n* = 20)	18 (90)	0 (0)	20 (100)	0 (0)	0 (0)
**Intima**					
All (*n* = 40)	33 (82.5)	0 (0)	40 (100)	0 (0)	0 (0)
Control (*n* = 20)	16 (80)	0 (0)	20 (100)	0 (0)	0 (0)
VV (*n* = 20)	17 (85)	0 (0)	20 (100)	0 (0)	0 (0)

Data are presented as number and percentage n (%). Abbreviations: cMGP, carboxylated matrix Gla protein; ucMGP, uncarboxylated matrix Gla protein; pMGP, phosphorylated matrix Gla protein; dpMGP, dephosphorylated matrix Gla protein; vK, von Kossa.
